# Efficacy and Safety of Deep Sedation and Anaesthesia for Complex Endoscopic Procedures—A Narrative Review

**DOI:** 10.3390/diagnostics12071523

**Published:** 2022-06-22

**Authors:** Daniela Godoroja-Diarto, Alina Constantin, Cosmin Moldovan, Elena Rusu, Massimilliano Sorbello

**Affiliations:** 1 Department Anaesthesia and Intennsive Care, Ponderas Academic Hospital, 014142 Bucharest, Romania; 2 Department Gastroenterology, Ponderas Academic Hospital, 014142 Bucharest, Romania; drconstantinalina@gmail.com; 3 Faculty of Medicine, University Titu Maiorescu, 040441 Bucharest, Romania; elenarusu98@yahoo.com; 4 Department of General Surgery, Hospital Clinic CF1 Witting, 010243 Bucharest, Romania; 5 Department Anaesthesia and Intennsive Care, AOU Policlinico San Marco, 95121 Catania, Italy; maxsorbello@gmail.com

**Keywords:** deep sedation, propofol, endoscopy

## Abstract

Propofol sedation for advanced endoscopic procedures is a widespread technique at present, which generates controversy worldwide when anaesthetic or non-anaesthetic personnel administer this form of sedation. There is some evidence for safe administered propofol sedation by non-anaesthetic personnel in patients undergoing endoscopy procedures, but there are only few randomised trials addressing the safety and efficacy of propofol in patients undergoing advanced procedures. A serious possible consequence of propofol sedation is the rapid and unpredictable progression from deep sedation to general anaesthesia mostly when elderly and frail patients are involved in the diagnosis or treatment of various neoplasia. This situation requires rescue measures with skilled airway management. The aim of this paper is to review the safety and efficacy aspects of sedation techniques, with special reference to propofol administration covering the whole patient journey, including preassessment, sedation options and discharge when advanced endoscopic procedures are performed.

## 1. Introduction

Advanced endoscopic procedures such as endoscopic retrograde cholangiopancreatography (ERCP) and endoscopic ultrasound, especially when fine-needle aspiration (EUS-FNA) is required, are uncomfortable procedures that require adequate sedation or general anaesthesia for termination successfully. Many of these interventions are accomplished worldwide. In the UK, every year, around 2 million procedures are performed each year, with 48,000 ERCP and many are recently registered in a national database [1]. The majority are performed under conscious sedation with benzodiazepines and opioids administered by the endoscopist. However, in prolonged and complex procedures, conscious sedation may provide inadequate patient or endoscopist comfort or ensue to patient oversedation, especially since high doses of benzodiazepines and opioids that may be used in addition to these advanced procedures may raise patient safety concerns with a negative impact on the patient’s outcome [2].

Propofol sedation for advanced endoscopic procedures has become widespread technique at present, which has generated controversy worldwide when anaesthetic or non-anaesthetic personnel administer this form of sedation. Monitored anaesthesia care (MAC), referred to as deep sedation, has been described as sedation and analgesia titrated to a level that preserves spontaneous breathing and airway reflexes [3] and has similar risks as general anaesthesia administered in a standard operating room (OR). Moreover, the evidence literature suggests that the anaesthesia risks in locations such as endoscopy suites are higher than in conventional ORs.

With an increasingly elderly population, the number of advanced endoscopic procedures, which are performed mostly for diagnosis, staging or treatment phases of different neoplasia is likely to increase, as is, accordingly, the requirement for adequate sedation. The aim of this paper is to review the safety and efficacy aspects of sedation techniques, with special reference to propofol sedation when advanced endoscopic procedures are performed. The key questions for the safety and efficacy of advanced endoscopic procedures refers to (1) requirement of different levels of sedation needed for performing advanced endoscopic procedures (conscious, deep sedation or general anaesthesia); (2) the place of procedure—endoscopy suite or operating theater—and the conditions required for a safe sedation; (3) administration of propofol by non-anaesthesia personnel or by anaesthetist, analysing this controversial area with the scope of acknowledging and implementation of the recommendations of the available guidelines. 

Consequently, our paper highlighted the risks of providing deep propofol sedation for interventional complex procedures, which are among the highest for sedated endoscopic procedure [4]. During these procedures, the sedation provider may experience a high intensity of pain stimulation, challenges in airway management that require specialised interventions, and a high incidence of hypoxemia with limited time available for recognition and management. Metzner et al. [5] analysed anaesthesia-related claims and concluded that 50% of all non-operating-room anaesthesia (NORA)-related deaths occurred during gastrointestinal endoscopy, and about half of these deaths were sedation-related. 

Hypoxemic events are more frequent with deep sedation, and cardiovascular events such as hypotension are more frequent with general anaesthesia. Factors responsible for hypotension are the decrease in systemic vascular resistance due to anaesthetic drugs, decrease in cardiac preload associated with positive-pressure ventilation and hypovolemia in some patients. In deep sedation, frequent coughing and hypoxemia may require withdrawal of the endoscope and completion of the procedure before the intended results are achieved. This is the reason that many patients need general anaesthesia to avoid the challenges involved in providing deep sedation for these advanced procedures. 

## 2. Safety of Sedation in Advanced Endoscopic Procedures

Sedation is defined by ASA as a continuum of states of consciousness from mild through to moderate sedation (conscious) to deep sedation, after which general anaesthesia follows Table 1 [2].

In patients undergoing ERCP procedures, deep propofol sedation is preferred to conscious sedation by gastroenterologists because often patients tolerate the procedure better [6,7]. A prospective study that reviewed 1550 ERCPs performed under conscious sedation with Midazolam and Demerol showed that patient cooperation under conscious sedation was not good in a significant number of patients, and this increased the difficulty of the procedure’s performance [6].

A Cochrane review that identified only four randomised studies comparing conscious sedation using midazolam and meperidine with propofol administered by non-anaesthesiologists for ERCP showed a faster and better recovery profile in propofol-sedation patients [7].

Deep sedation, as defined by the ASA, may require airway management, and spontaneous ventilation may be inadequate to maintain oxygenation and gas exchange. Depending on the amount of drug used and the sensitivity of the patients, the level of sedation can fluctuate easily, and many patients under conscious sedation may progress unintentionally to a level of deep sedation. If this happens when the sedation provider is not trained in airway management, there can be many adverse events, which are sometimes dangerous for patients [4].

Propofol is a short-acting intravenous anaesthetic agent and has been shown to provide better sedation and recovery quality, improve patient and endoscopist satisfaction, and the overall outcome of the procedural sedation compared to standard conscious sedation [8,9]. However, the use of propofol has been extensively debated. This is due to its narrow therapeutic index, with a high pharmacologic variability, calculated at 300–400% [10], the lack of an antidote and the risk of cardiorespiratory complications, especially in old patients.

A small increase in dosage when further amplified by the use of opioid adjuvants may cause the patient to switch from deep sedation to an unpredictable state of general anaesthesia. If we administer too little it can trigger cough or laryngospasm, while too much can lead to apnoea and both can cause hypoxemia. Therefore, suboptimal subsedation or oversedation can lead to complications, and in rare cases to disastrous consequences mostly in patients with unprotected airways. In the case of general anaesthesia, the airways are protected and the results of variability in the effect of the drug are not a concern. because a trained anaesthetist will be able to anticipate this variability and prevent or treat hypoxemia. Sedation provided by a dedicated anaesthesia team for all endoscopies, especially when complex, increases the safety and efficiency of procedures.

This is the main reason that the Royal College of Anaesthetists in the UK recommends that patients undergoing deep sedation receive the same level of care as those under general anaesthesia [11]. 

## 3. Choice of Drugs and Airway Management

Propofol is the best choice for its desired properties compared to all other sedatives used during endoscopy. Intravenous conscious sedation usually uses a combination of benzodiazepines, such as midazolam, and a short-acting opioid, such as fentanyl. The combination is safe and acceptable for diagnostic endoscopy and colonoscopy, but is unsuitable for patient and endoscopist satisfaction in complex endoscopic procedures.

Another sedative, dexmedetomidine, an alpha-2 adrenergic receptor, which is safe because it did not produce respiratory depression, has an unacceptably long speed of onset [12]. 

A newer benzodiazepine remimazolam that has been shown as a better choice for procedural sedation than midazolam [13] is under investigation in a randomised trial that actually still recruits patients. It is compared with the safety and efficacy of association remimazolam alfentanil with the association of propofol-alfentanil for moderate-deep sedation in ERCP patients [14]. 

Other technique for deep sedation in ERCP is combination of ketamine and propofol that reduces the dosage of individual drugs, thereby providing a better safety and satisfaction profile than the combination with an opioid (alfentanil) during ERCP [15].

A well-known technique for deep sedation is continuous propofol infusion with or without target controlled infusion combined with a short-acting opioid (fentanyl or remifentanil) [16].

Considering the advantages and limitations of the drugs that are currently available, it is essential to adapt many techniques to the sedation process and treat all undesirable consequences.

The safety of one or another technique can be ensured by the following airway-management manoeuvres: chin lift and jaw thrust to open the obstructed upper airway; in patients with low pulmonary reserve, obstructive sleep apnoea or severe obesity a low threshold for tracheal intubation should be considered [17]; a face mask and portable ventilation devices should be readily available and the provider should know how to use them. The anaesthesiologist should anticipate and be actively involved in the process of sedation. 

## 4. Complications

The most important complications in patients undergoing advanced endoscopic procedures are hypoxemia, hypotension and aspiration, and the most important factors that can increase the severity of these events are ASA status, hypovolemia, oxygenation status and the monitoring techniques used during the procedure. However, aspiration has rarely been reported as an adverse event in endoscopic procedures because patients are usually fasted, but prolonged or difficult procedures may still be associated with an increased risk of regurgitation and aspiration [18].

In 2005 British Society of Gastroenterologists performed a survey reviewing the safety of performing ERCP under conscious sedation [1,19] and identified a procedure-related mortality rate of 0.4% over a large group of patients.

In a retrospective study, Wehrmann at al. reviewed 3937 ERCP, 236 EUS and many OGD over a 6-year period. Assisted ventilation was necessary in 40 patients (0.4%); 9 patients required endotracheal intubation (0.09); and 4 patients died, emergency endoscopic examinations and a total propofol dose >100 mg being found as independent risk factors for adverse events [20].

Another study on 200 patients who underwent ERCP suggests that anaesthesiologist-administered sedation is safe in those patients and is associated with a high rate of successful ERCP with only minor events reported that did not require termination of the procedure in advance, shorter procedural time with fast recovery and high patient and endoscopist satisfaction [21].

Cot’e et al. [4] analysed in a prospective study of 799 cases of patients with complex endoscopy procedures under deep propofol sedation and found a hypoxemia rate of 12.8% and a hypotension rate of 0.8% during the procedures. The failed-procedure rate was 0.6%. More than 60% of patients had an ASA III or higher. Airway manoeuvres were required in 14.4% of cases, and the independent predictors for these interventions were ASA III or higher and increased BMI.

Four randomised trials compared conscious sedation with midazolam and demerol to propofol sedation [22,23,24,25] for ERCP procedures performed by non-anaesthetic providers. Two of the trials included ASA I–III patients [22,24], while the other two also included ASA IV patients [23,25] with zero immediate mortality rate for both techniques. In one study, the procedure failed because of the sedation technique in five patients [25], but in the other three trials, sedation was successful in all patients. Outcomes such as hypoxemia and hypotension (systolic < 90 mm Hg) were not significant different for both sedation techniques, in all studies. However, in the two studies where patients did not receive oxygen during the procedure, a high incidence of hypoxemia was reported for both sedation techniques [23,25].

ProSed2 study showed that the risk of complications was linearly associated with the duration of procedure, with the odd ratio for complications increasing by 1.8 for procedures less than 10 min, compared with 7.9 for procedures longer than one hour [26]. The procedure subtypes’ comparison in the same study showed an increased risk for ERCP and therapeutic EUS versus diagnostic EUS.

## 5. Selection of Anaesthesia Technique Based on Comorbidity and Procedure

The safest sedation technique is obtained when the provider adapts sedation to the patient’s needs, although propofol sedation has shown a significantly better recovery profile than conscious sedation for ERCP procedures in all present—but a limited number of—studies [22,23]. Patients range from the American Society of Anaesthesiology (ASA) physical classification I–IV [27], but many of them presenting for advanced endoscopic procedures, including ERCP, may often be elderly and ill, with a significant proportion in the ASAIII and IV categories. This group of patients may be prone to cardiorespiratory complications, and the elderly are more prone to aspiration due to more depressed airway reflexes. Consequently, in this group of patients, special care must be taken to ensure adequate volume repletion and oxygenation before the procedure beginning. The deep sedation should be performed by anaesthetists considering a low threshold for endotracheal intubation with general anaesthesia. 

In elderly and frail patients, some authors recommend target controlled anaesthesia (TCI) using short-acting drugs (propofol and remifentanil) that shorten the duration of anaesthesia [28,29]. TCI, compared to the bolus or continuous infusion technique, provides stable control of anaesthesia with the possibility of maintaining spontaneous ventilation and ensures hemodynamic stability with a faster recovery [29,30] proving to be the anaesthesia of choice for this category of patients.

Due to EUS-guided fine-needle aspiration (FNA) or fine-needle biopsy (FNB) [31], in pancreaticobiliary diseases a number of advanced EUS-guided procedures are increasingly performed in practice, such as EUS-guided drainage [32] or tumour ablation [33]. Therefore, advanced EUS procedures are more time-consuming, more painful and lengthier than the diagnostic procedures, and deep sedation or general anaesthesia is required to meet EUS quality requirements.

## 6. Non-Anaesthesiologist Providers

Internationally, sedation with propofol in endoscopy by non-anaesthesiologists is widespread but also intensely controversial and debated. Although in the UK gastroenterologists typically perform ERCP under conscious sedation, both the Royal College of Anaesthetists (UK) and the American Society of Anaesthesiologists consider that propofol sedation should ideally be administered by anaesthetic staff [11]. Providing anaesthetist-led deep sedation is a challenge facing endoscopy units around the world because of obstacles such as out-of-demand anaesthesia, funding and staffing [34].

Propofol is a valuable sedative in advanced endoscopic procedures and its use has increased in recent years due to its favourable pharmacokinetic profile compared to traditional conscious sedation [35]. Although propofol is generally administered by anaesthesiologists, there is growing evidence to suggest that endoscopists may safely administer or supervise the administration of propofol sedation without the involvement of an anaesthetist [36].

Several studies showed that sedation with propofol by non-anaesthesiologists has been associated with shorter recovery times compared to standard moderate sedation [37,38], thus supporting its routine use. Nearly half a million cases have been reported, with a low incidence of adverse events [39]. The American and Canadian Society of Gastroenterologists have endorsed the use of propofol by non-anaesthetic staff [8]. Additionally, some meta-analyses of several randomised controlled trials reported similar rates of adverse events between sedation with propofol or traditional sedative agents, whether administered by anaesthetists or non-anaesthetic personnel [40,41].

A recent multicentre German study on sedation-related complications (ProSed 2) analysed more than 300,000 patients undergoing endoscopy and found that while overall sedation-related complications were generally low (0.01%), the lowest rate was in patients receiving propofol alone [26].

The administration of propofol by anaesthesia specialists significantly increases the costs of endoscopic procedures without a recognised improvement in the results of safety and efficacy [35,37]. Conversely, Anaesthesia Societies claim that administration of propofol by non-anaesthesiology providers is unsafe, stating that propofol should be given only by people trained in general anaesthesia [41,42]. The US Food and Drug Administration in 2010 rejected a 2005 petition by the American Society of Gastroenterologists to remove the requirement that "for general anaesthesia or monitored anaesthesia care (MAC), propofol should only be administered by individuals trained in the administration of general anaesthesia and not involved in the surgical/diagnostic procedure” [43].

Despite these warnings, and although the current guidelines of the American Society of Gastrointestinal Endoscopy (ASGE) recommend the use of sedation by non-anaesthesiology providers in the presence of appropriate specialised training, selection of patients, and staff dedicated to continuous physiological monitoring [35], regulations regarding the administration of propofol are still highly variable and determined by regional and local rules. As a result, the practice of non-anaesthesiology providers is quite limited in several countries, including Romania [44,45].

These controversial studies have shown that general anaesthesia administered by an anaesthetist is associated with a significantly higher EUS-FNA diagnostic efficiency of a solid pancreatic mass [46] versus the support for non-anaesthesiology providers of sedation with propofol during the EUS-FNA or diagnostic EUS [47]. The development of proficiency in propofol sedation for non-anaesthesia personnel administering propofol sedation for patients undergoing advanced endoscopic procedures can be acceptable for non-anaesthesia personnel if they have been adequately trained and the safety recommendations of actual guidelines are accomplished.

## 7. Anaesthetic Guidelines

The Royal College of Anaesthetists (UK), in association with the British Society of Gastroenterology, have issued guidelines for administration of propofol sedation for complex procedures [34]. This position statement outlines the indications for anaesthetist-led deep sedation using propofol or general anaesthesia for complex gastrointestinal endoscopy and recommendations related to three important directions: pre-anaesthetic assessment, local standard operating procedures and training.

Preprocedural anaesthetic assessment should reduce the risk for all patients and identify high-risk patients. 

The choice of sedation should be determined by several factors.

Patient-related factors such as ASA III or higher, old age, comorbidities, difficult airway, obesity and obstructive sleep apnoea (OSA) are identified as risk factors for cardiac and respiratory complications during endoscopy. In a retrospective study of more than 1 million patients undergoing endoscopy and colonoscopy, the higher American Society of Anaesthesiologist’s (ASA) class was associated with an increased risk of adverse events [48].These guidelines also recommend that all patients considered for sedation are screened for OSA. The STOP-BANG questionnaire is a simple and reliable score that can diagnose and grade the severity of obstructive sleep apnoea (OSA) [49,50,51].

Procedural-related factors are considered to be a poor tolerance of prior endoscopy under conscious sedation, duration of the procedure and type of procedure. Therefore, propofol deep sedation should be considered for (but not limited to) ERCP, therapeutic EUS and any prolonged therapeutic procedure. Emergency procedures are recommended for general anaesthesia with tracheal intubation, especially when risk of aspiration is present, such as the presence of significant gastrointestinal bleeding, recent oral intake or during the procedure, e.g., trans-gastric endoscopic drainage of pancreatic fluid collection. The decision on deep sedation or general anaesthesia will be made as part of the anaesthesia pre-assessment process and will be affected by local protocols and operator experience.

The second recommendation is related to a local standard operating procedure to ensure that minimum standards of endoscopy room, staffing and the delivery of all types of deep sedation and anaesthesia are kept. Standards of endoscopy room requirements are continuous-flow oxygen, an anaesthetic machine with medical gases and vacuum systems, immediate access to the resuscitation trolley, defibrillator and equipment for tracheal intubation. Basic monitoring includes electrocardiography, oxygen saturation and noninvasive blood pressure, but capnography monitors and bispectral index monitors with target-control infusion pumps are recommended.

The third recommendation refers to training that should be included as an essential competency for endoscopy trainees when referring to conscious sedation for endoscopy, or for anaesthetic trainees when referring to deep sedation and anaesthesia for endoscopy.

Guidelines for sedation and anaesthesia in GI endoscopy published by the American Society for Gastrointestinal Endoscopy (ASGE) [35] consider ASA task force guidelines for sedation and analgesia administered by non-anaesthesiologists [52]. According to the ASA guidelines for sedation the likelihood of sedation-related adverse events is increased by the presence of one or more sedation-related risk factors together with the probability for deep sedation. Furthermore, an anaesthesia professional should be considered in prolonged or therapeutic endoscopic procedures requiring deep sedation, anticipated intolerance to standard sedatives, severe cardiac or respiratory comorbidities (ASA class IV or V), and increased risk of airway obstruction (obesity, OSA, difficult airway), and general anaesthesia with controlled ventilation should be planned. 

In the same guidelines, the minimal patient-monitoring requirements for sedation in endoscopic procedures are noninvasive blood pressure, heart rate, pulse oximetry [53,54] the visual assessment of ventilatory activity, level of consciousness and discomfort [54]. ASA guidelines recommend electrocardiogram (ECG) monitoring in patients with significant cardiovascular disease or arrhythmia, significant pulmonary disease, the elderly and in anticipated prolonged procedures [52].

The ASA and the ASGE recommend the use of supplemental oxygen for moderate sedation, and set this as mandatory for all procedures with deep sedation [52,55]. Routine administration of supplemental oxygen has been shown to reduce oxygen desaturation during sedation for endoscopic procedures [56]. 

Capnography measures the partial pressure of carbon dioxide during the respiratory cycle and can quickly detect disordered or depressed respiratory activity [57]. Capnography has been shown to detect depressed respiratory activity prior to transient hypoxemia emergence [58,59,60]. In this study, independent risk factors for hypoxemia were age, high body mass index, sleep apnoea and high doses of sedatives. However, in conscious sedation, it seems that capnography does not bring benefits for the prevention of hypoxemia [61].

Postprocedural monitoring should be continued after completion of the procedure for the adverse events and patients discharged according to standardised criteria assessing recovery after sedation.

Sedation with propofol and anaesthesia experience for advanced endoscopic procedures (EUS, EUS-FNA and ERCP at Ponderas Academic Hospital, Bucharest, Romania.

At our institution, propofol sedation or general anaesthesia for complex procedures such as ERCP and EUS or EUS-FNA procedures are administered by specialist anaesthesiologists in the operating theater. 

All patients received supplemental oxygen during the procedure. All patients were continuously monitored for heart rate (using a three-lead electrocardiogram), blood pressure (using an automated blood-pressure cuff and serial measurements every 5 min), oxygen saturation (by pulse oximetry) and capnography. Capnography was ensured with the Capnostream™ 20p patient monitor with Microstream™ technology using Microstream™ Advance filter lines with nasal-only CO_2_ sampling, as well as the supplemental oxygen-delivery system (manufactured by Medtronic) or using a capnograph from an anaesthetic machine attached to an oxygen mask covering only the nose.

The capnograph trace from this device is highly reliable, and we found that it was invaluable for maintaining appropriate sedation levels in the patient and detecting respiratory depression or obstruction. 

Vascular access was secured in all patients via the peripheral vein and an infusion of 0.9% saline was administered. 

For deep sedation, we used propofol in bolus intravenous administration, continuous infusion or TCI. In the TCI mode, propofol was started at 10 mg/mL with the Schnider effect model, with an initial target of 0.5 μg/mL.

For general anaesthesia with intubation, after 3 min of pre-oxygenation with 100% oxygen via a tight-fitting facemask and an expired oxygen fraction superior to 92%, anaesthesia was induced using two methods. In our clinic, patients are treated according to a low-opioid anaesthesia protocol using adjuvants such as magnesium sulphate 40 mg kg^−1^ LBW, lidocaine 1.5 mg kg^−1^ LBW, dexamethasone 10 mg and ketamine 20 mg [62].

The method of anaesthesia used was in accordance with patient particularities and anaesthetist preferences. General balanced anaesthesia on inhalator gas: induction was performed by fentanyl (1–2 μg/kg) and propofol (1.5–2 mg/kg) and maintenance of anaesthesia was achieved with sevoflurane at 1.0–1.5 age-adjusted MAC and a 50% mix of oxygen and air. TCI: induction and maintenance were carried out with propofol and remifentanil. In this group of patients, we monitored the depth of anaesthesia using the Bispectral Index™ (BIS™) Monitoring System.

For induction, TCI propofol 10 mg/mL was started with the Schnider effect model, with an initial target 3 μg/mL for intubated patients. Remifentanil (50 μg/mL), was administered by the Minto effect model with an initial target of 0.75 ng/mL. Anaesthesia was maintained by the same drugs in TCI mode. 

In all intubated patients, muscle relaxation was achieved with rocuronium 0.6 mg/kg. Variations of less than 30% of the mean arterial pressure (MAP) and heart rate (HR) were considered hemodynamic objectives.

In Ponderas Academic Hospital, Bucharest Romania, from January 2017 to April 2022, 500 cases of EUS were performed; out of them, there were 193 EUS-FNA and 200 ERCP. Deep sedation with propofol with spontaneous breathing was performed on 403 patients with EUS, and general anaesthesia with elective intubation on 297 patients, including all ERCP patients. The baseline characteristics of the series of 700 patients who underwent EUS procedures and ERCP at our centre are reported in Table 2. 

Continuous variables are reported as mean values and standard deviations. Abbreviations: ASA, American Society of Anaesthesiology; EUS, endoscopic ultrasound; FNA, fine-needle aspiration; FNB, fine-needle biopsy; GA = general anaesthesia ERCP = endoscopic retrograde cholangiopancreatography.These are the results of our yet unpublished retrospective data, where we had a very low transient hypoxemia rate and a low transient hypotension rate. Sedation was successful in all the patients. Our indications for intubation with general anaesthesia were ERCP, ASA 3 or more, severe obesity with OSA, severe respiratory disease, anticipated prolonged procedure and history of severe regurgitation. No patients had to be intubated as an emergency. Further observational prospective series or randomised controlled trials (RCTs) will confirm these findings. 

## 8. Conclusions

Sedation for advanced endoscopic procedures is a challenging area where there is an overlap between anaesthetic and non-anaesthetic providers. The requirement for anaesthetist-led deep sedation with propofol worldwide for complex gastrointestinal endoscopy is increasing. However, propofol can be administered safely by non-anaesthetic personnel, even for advanced endoscopic procedures, to reduce cost or in low-resource settings or unavailability of an anaesthesia specialist. 

The guidelines released highlight the challenges faced and provide a framework for anaesthetic and non-anaesthetic personnel to perform the procedural sedation with the following recommendations:

1. Pre-anaesthetic assessment of all patients undergoing endoscopic procedures to evaluate the risk of sedation related to pre-existing pathology.
2. Non-anaesthesiology providers must have specific training in the sedation administration for endoscopy and have the skills to diagnose and manage sedation-related complications, especially when unintended deep sedation occurs or rescue of airway associated events.
3. Routine monitoring of blood pressure, oxygen saturation, heart rate and clinical observation during all endoscopic procedures using sedation.
4. Supplemental oxygen administration considered for moderate and deep sedation.
5. Capnography monitoring considered for patients undergoing deep sedation.
6. Sedation administered by anaesthesia provider considered for complex endoscopic procedures or patients with important comorbidities or at risk for airway compromise.

The paucity of data available to guide anaesthesiologists in selecting between deep propofol sedation and general anaesthesia/intubation for advanced procedures or frail patients, the challenges of providing anaesthesia in remote locations (unfamiliarity with the environment, lack of immediate access to all the equipment, medications and support personnel) suggest that further prospective studies are needed to evaluate the effect of experience and comfort of anaesthesiologists in determining their choice and to evaluate when sedation is provided by an anaesthetist or non-anaesthetist.

The review undoubtedly has limitations, particularly the nonstandardised method for the selection of studies participating, which might have contributed to a possible selection bias. 

Communication between endoscopist and anaesthesia provider about the expected complexity of the procedure, estimated time to perform it, together with any known gastrointestinal issues should contribute to decision making for the procedure performance.

A future without significant anaesthesia involvement in this controversial area is difficult to foresee, but cooperation and discussion between gastroenterologists and anaesthesiologists may lead to a reliable solution for the safety of the patients and efficacy of procedure.

## Figures and Tables

**Table 1:** Levels of sedation and anaesthesia.

Minimal Sedation (Anxiolysis)		Moderate Sedation (Conscious Sedation)	Deep Sedation	General Anaesthesia
**Responsiveness**	Normal response to verbal stimulation	Purposeful response to verbal or tactile stimulation	Purposeful response after repeated or painful stimulation	Unarousable even with painful stimulus
**Airway**	Unaffected	No intervention required	Intervention may be required	Intervention often required
**Spontaneous ventilation**	Unaffected	Adequate	May be inadequate	Frequently inadequate
**Cardiovascular function**	Unaffected	Usually maintained	Usually maintained	May be impaired

Continuum of Depth of Sedation Definition of General Anesthesia and Levels of Sedation/Analgesia (Developed by the American Society of Anesthesiologists) (Approved by ASA House of Delegates on 13 October 1999) [2].

**Table 2:** Baseline patients’ characteristics.

Variable		N	Deep Sedation	GA	Gender		Age (Years)	ASA ≤ 2
**Procedure**					M	F		
**EUS**		500	403	97	276	224	59 ± 9	389
			80.%	19.4%	55.2%	44.8%		77.8%
	**EUS-**	193	145	48				
	**FNA/FNB**	38.6%						
	**EUS-**	307	258	49				
	**diagnostics**	61.4%						
**ERCP**		200	0	200	103	97	63 ± 7	128
				(100%)	51.5%	48.5%		64%

## Data Availability

Not applicable.

## References

[1] Lee T.J., Siau K., Esmaily S., Docherty J., Stebbing J., Brookes M.J., Broughton R., Rogers P., Dunckley P., Rutter M.D. (2019). Development of a national automated endoscopy database: The United Kingdom National Endoscopy Database (NED). United Eur. Gastroenterol. J..

[2] Dunckley P., Rutter M.D. (1999). ASA “Continuum of Depth of Sedation, Definition of General Anaesthesia, and Levels of Sedation/Analgesia”, ASA Standards, Guidelines and Statements. https://www.asahq.org/standards-and-guidelines/continuum-of-depth-of-sedation-definition-of-general-anesthesia-and-levels-of-sedationanalgesia.

[3] ASA Position on Monitored Anesthesia Care Last Amended: 17 October 2018 (Original Approval: 25 October 2005). https://www.asahq.org/standards-and-guidelines/continuum-of-depth-of-sedation-definition-of-general-anesthesia-and-levels-of-sedationanalgesia.

[4] Coté G.A., Hovis R.M., Ansstas M.A., Waldbaum L., Azar R.R., Early D.S., Edmundowicz S.A., Mullady D.K., Jonnalagadda S.S. (2010). Incidence of sedation related complications with propofol use during advanced endoscopic procedures. Clin. Gastroenterol. Hepatol..

[5] Metzner J., Posner K.L., Domino K.B. (2009). The risk and safety of anesthesia at remote locations: The US closed claims analysis. Curr. Opin. Anaesthesiol..

[6] Penston J., Southern P., Penston P., Daws D. (2009). ERCP in a district general hospital in England: A review of 1550 procedures over nine years. Internet J. Gastroenterol..

[7] Garewal D., Powell S., Milan S.J., Nordmeyer J., Waikar P. (2012). Sedative techniques for endoscopic retrograde cholangiopancreatography. Cochrane Database Syst. Rev..

[8] McQuaid K.R., Laine L. (2008). A systematic review and meta-analysis of randomized, controlled trials of moderate sedation for routine endoscopic procedures. Gastrointest. Endosc..

[9] Smith I., Durkin D., Lau K.W., Hebbar S. (2018). Establishing an anaesthetist delivered propofol sedation service for advanced endoscopic procedures: Implementing the RCA/BSG guidelines. Frontline Gastroenterol..

[10] Vuyk J. (1998). TCI: Supplementation and drug interactions. Anaesthesia.

[11] The Joint Royal Colleges of Anaesthetists (RCoA), British Society of Gastroenterology (BSG) Working Party (2011). Guidance for the Use of Propofol Sedation for Adult Patients Undergoing Endoscopic Retrograde Cholangiopancreatography (ERCP) and Other Complex Upper GI Endoscopic Procedures. https://www.bsg.org.uk/wp-content/uploads/2019/12/Guidance-for-the-use-of-propofol-sedation-for-adult-patients-undergoing-Endoscopic-Retrograde-Cholangiopancreatography-ERCP-and-other-complex-upper-.pdf.

[12] Amornyotin S. (2016). Dexmedetomidine in gastrointestinal endoscopic procedures. World J. Anesthesiol..

[13] Jhuang B.-J., Yeh B.-H., Huang Y.-T., Lai P.-C. (2021). Efficacy and Safety of Remimazolam for Procedural Sedation: A Meta-Analysis of Randomized Controlled Trials With Trial Sequential Analysis. Front. Med..

[14] Ianbo Y. Efficacy and Safety Profile of Remimazolam-Alfentanil Combination for ERCP Sedation. https://clinicaltrials.gov/ct2/show/NCT04658173.

[15] Eberl S., Koers L., van Hooft J., de Jong E., Hermanides J., Hollmann M.W., Preckel B. (2020). The effectiveness of a low-dose esketamine versus an alfentanil adjunct to propofol sedation during endoscopic retrograde cholangiopancreatography: A randomised controlled multicentre trial. Eur. J. Anaesthesiol..

[16] García Guzzo M.E., Fernandez M.S., Sanchez Novas D., Salgado S.S., Terrasa S.A., Domenech G., Teijido C.A. (2020). Deep sedation using propofol target-controlled infusion for gastrointestinal endoscopic procedures: A retrospective cohort study. BMC Anesthesiol..

[17] Godoroja D., Sorbello M., Margarson M. (2019). Airway management in obese patients: The need for lean strategies. Trends Anaesth. Crit. Care.

[18] Green S.M., Leroy P.L., Roback M.G., Irwin M.G., Andolfatto G., Babl F.E., Barbi E., Costa L.R., Absalom A., Carlson D.W. (2020). An international multidisciplinary consensus statement on fasting before procedural sedation in adults and children. Anaesthesia.

[19] (2004). Scoping Our Practice. https://www.ncepod.org.uk/2004sop.html.

[20] Wehrmann T., Riphaus A. (2008). Sedation with propofol for interventional endoscopic procedures: A risk factor analysis. Scand. J. Gastroenterol..

[21] Zhang C.C., Ganion N., Knebel P., Bopp C., Brenner T., Weigand M.A., Sauer P., Schaible A. (2020). Sedation-related complications during anaesthesiologist-administered sedation for endoscopic retrograde cholangiopancreatography: A prospective study. BMC Anesthesiol..

[22] Riphaus A., Stergiou N., Wehrmann T. (2005). Sedation with propofol for routine ERCP in high-risk octogenarians: A randomized, controlled study. Am. J. Gastroenterol..

[23] Vargo J., Zuccaro G., Dumot J., Shermock K., Morrow J.B., Conwell D., Trolli P., Maurer W. (2002). Gastroenterologist administered propofol versus midazolam/meperidine for advanced upper endoscopy: A propective randomised trial. Gastroenterology.

[24] Schilling D., Rosenbaum A., Schweizer S., Richter H., Rumstadt B. (2009). Sedation with propofol for interventional endoscopy by trained nurses in high-risk octogenarians: A prospective, randomized, controlled study. Endoscopy.

[25] Kongkam P., Rerknimitr R., Punyathavorn S., Sitthi-Amorn C., Ponauthai Y., Prempracha N., Kullavanijaya P. (2008). Propofol infusion versus intermittent meperidine and midazolam injection for conscious sedation in ERCP. J. Gastrointest. Liver Dis..

[26] Behrens A., Kreuzmayr A., Manner H., Koop H., Lorenz A., Schaefer C., Plauth M., Jetschmann J.-U., von Tirpitz C., Ewald M. (2019). Acute sedation-associated complications in GI endoscopy (ProSed 2 Study): Results from the prospective multicentre electronic registry of sedation-associated complications. Gut.

[27] American Society of Anesthesiologists ASA Physical Status Classification System. http://www.asahq.org/resources/clinical-information/asa-physical-status-classification-system.

[28] Gillham M.J., Hutchinson R.C., Carter R., Kenny G.N. (2001). Patient-maintained sedation for ERCP with a target controlled infusion of propofol: A pilot study. Gastrointest. Endosc..

[29] Fanti L., Agostoni M., Casati A., Guslandi M., Giollo P., Torri G., Testoni P.A. (2004). Target-Controlled propofol infusion during monitored anesthesia in patients undergoing ERCP. Gastrointest. Endosc..

[30] Billard V. (2009). Target controlled infusion of opioid in clinical practice. Pract. Anesth. Reanim..

[31] Facciorusso A. (2020). Endoscopic ultrasound-guided tissue sampling of pancreatic lesions. Minerva Gastroenterol. Dietol..

[32] Rodrigues-Pinto E., Baron T.H. (2016). Evaluation of the AXIOS stent for the treatment of pancreatic fluid collections. Expert Rev. Med. Devices.

[33] Facciorusso A., Del Prete V., Antonino M., Buccino V.R., Muscatiello N. (2019). Response to repeat echoendoscopic celiac plexus neurolysis in pancreatic cancer patients: A machine learning approach. Pancreatology.

[34] Sidhu R., Turnbull D., Newton M., Thomas-Gibson S., Sanders D.S., Hebbar S., Haidry R.J., Smith G. (2019). Webster G-Deep sedation and anaesthesia in complex gastrointestinal endoscopy: A joint position statement endorsed by the British Society of Gastroenterology (BSG), Joint Advisory Group (JAG) and Royal College of Anaesthetists (RCoA). Frontline Gastroenterol..

[35] Early D.S., Lightdale J.R., Vargo J.J., Acosta R.D., Chandrasekhara V., Chathadi K.V., Evans J.A., Fisher D.A., Fonkalsrud L., Hwang J.H. (2018). Guidelines for sedation and anesthesia in GI endoscopy. Gastrointest. Endosc..

[36] Rex D.K., Deenadayalu V.P., Eid E., Imperiale T.F., Walker J.A., Sandhu K., Clarke A.C., Hillman L.C., Horiuchi A., Cohen L.B. (2009). Endoscopist-Directed Administration of Propofol: A Worldwide Safety Experience. Gastroenterology.

[37] Cohen L.B., Hightower C.D., Wood A.D., Miller K.M., Aisenberg J. (2004). Moderate level sedation during endoscopy: A prospective study using low-dose propofol, meperidine/fentanyl, and midazolam. Gastrointest. Endosc..

[38] Hassan C., Rex D.K., Cooper G.S., Benamouzig R. (2012). Endoscopist-directed propofol administration versus anesthesiologist assistance for colorectal cancer screening: A cost-effectiveness analysis. Endoscopy.

[39] Byrne M.F., Chiba N., Singh H., Sadowski D.-H. (2008). Propofol use for sedation during endoscopy in adults: A Canadian association of gastroenterology position statement. Can. J. Gastroenterol..

[40] Singh H., Poluha W., Cheang M., Choptain N., Inegbu E., Baron K., Taback S.P. (2008). Propofol for sedation during colonoscopy. Cochrane Database Syst. Rev..

[41] American Society of Anesthesiologists (ASA) AANA-ASA Joint Statement Regarding Propofol Administration. https://www.asahq.org/standards-and-guidelines/statement-of-grantingprivileges-for-administration-of-moderate-sedation-to-practitioners.

[42] American Society of Anesthesiologists (ASA) ASA House of Delegates Statement on Safe Use of Propofol. https://www.asahq.org/standards-and-guidelines/statement-of-granting-privileges-foradministration-of-moderate-sedation-to-practitioners.

[43] Department of Health and Human Services (2010). Petition Denial for Request for Removal of Warning of Labeling for Diprivan (Propofol), Docket no FDA-2005-P-0059. https://digestivehealth.net/images/FDA-2005-P-0059-0080_1_.pdf.

[44] Nishizawa T., Suzuki H. (2018). Propofol for gastrointestinal endoscopy. United Eur. Gastroenterol. J..

[45] Conigliaro R., Fanti L., Manno M., Brosolo P. (2017). Italian Society of Digestive Endoscopy (SIED) position paper on the non-anaesthesiologist administration of propofol for gastrointestinal endoscopy. Dig. Liver Dis..

[46] Ootaki C., Stevens T., Vargo J., You J., Shiba A., Foss J., Borkowski R., Maurer W. (2012). Does General Anesthesia Increase the Diagnostic Yield of Endoscopic Ultrasound-guided Fine Needle Aspiration of Pancreatic Masses?. Anesthesiology.

[47] Facciorusso A., Turco A., Barnabà C., Longo G., Dipasquale G., Muscatiello N. (2020). Efficacy and Safety of Non-Anesthesiologist Administration of Propofol Sedation in Endoscopic Ultrasound: A Propensity Score Analysis. Diagnostics.

[48] Enestvedt B.K., Eisen G.M., Holub J., Lieberman D.A. (2013). Is the American Society of Anesthesiologists classification useful in risk stratification for endoscopic procedures?. Gastrointest. Endosc..

[49] Corso R.M., Piraccini E., Agnoletti V., Lippi M., Buccioli M., Negro A., Gambale G., Ricci E. (2012). Clinical use of the STOP-BANG questionnaire in patients undergoing sedation for endoscopic procedures. Minerva Anestesiol..

[50] Godoroja D. (2017). Development and Feasibility of Two Novel Scores (DX-OSA and A-OSA) for the Identification of Significant Obstructive Sleep Apnoea in Surgical Patients with Obesity. J. Anesth. Clin. Res..

[51] Di Lorenzo N., Antoniou S.A., Batterham R., ·Busetto L. (2020). Godoroja D-Clinical practice guidelines of the European Association for Endoscopic Surgery (EAES) on bariatric surgery: Update 2020 endorsed by IFSO-EC, EASO and ESPCOP. Surg. Endosc..

[52] American Society of Anesthesiologists Task Force on Sedation and Analgesia by Non-Anesthesiologists (2002). Practice guidelines for sedation and analgesia by non-anesthesiologists. Anesthesiology.

[53] Waring J.P., Baron T.H., Hirota W.K., Goldstein J.L., Jacobson B.C., Leighton J.A., ShawnMallery J., Faigel D.O. (2003). Guidelines for conscious sedation and monitoring during gastrointestinal endoscopy. Gastrointest. Endosc..

[54] Calderwood A.H., Chapman F.J., Cohen J., Cohen L.B., Collins J., Day L.W., Early D.S. (2014). Guidelines for safety in the gastrointestinal endoscopy unit. Gastrointest. Endosc..

[55] Vargo J.J., DeLegge M.H., Feld A.D., Gerstenberger P.D., Kwo P.Y., Lightdale J.R., Rex D.K., Schiller L.R. (2012). Multisociety sedation curriculum for gastrointestinal endoscopy. Gastrointest. Endosc..

[56] Bell G.D., Morden A., Bown S., Coady T., Logan RF A. (1987). Prevention of hypoxaemia during upper-gastrointestinal endoscopy by means of oxygen via nasal cannulae. Lancet.

[57] Gerstenberger P.D. (2010). Capnography and patient safety for endoscopy. Clin. Gastroenterol. Hepatol..

[58] Lightdale J.R., Goldmann D.A., Feldman H.A., Newburg A.R., DiNardo J.A., Fox V.L. (2006). Microstream capnography improves patient monitoring during moderate sedation: A randomized, controlled trial. Pediatrics.

[59] Qadeer M.A., Vargo J.J., Dumot J.A., Lopez R., Trolli P.A., Stevens T., Sanaka M.R., Zuccaro G. (2009). Capnographic monitoring of respiratory activity improves safety of sedation for endoscopic cholangiopancreatograph and ultrasonography. Gastroenterology.

[60] Friedrich-Rust M., Welte M., Welte C., Albert J., Meckbach Y., Herrmann E., Kannengiesser M., Trojan J., Filmann N., Schroeter H. (2014). Capnographic monitoring of propofol-based sedation during colonoscopy. Endoscopy.

[61] Mehta P.P., Kochhar G., Albeldawi M., Kirsh B., Rizk M., Putka B., John B., Wang Y., Breslaw N., Lopez R. (2016). Capnographic monitoring in routine EGD and colonoscopy with moderate sedation: A prospective, randomized, controlled trial. Am. J. Gastroenterol..

[62] Godoroja D., Hainarosie D., Zaharencu A., Copaescu C. (2019). Portal Vein Thrombosis a Rare but Life-threatening Complication after Laparoscopic Sleeve Gastrectomy: A 5 Years Study in a Bariatric Center of Excellence. Chirurgia.

